# Fabrication of NaYF_4_:Yb,Er Nanoprobes for Cell Imaging Directly by Using the Method of Hydrion Rivalry Aided by Ultrasonic

**DOI:** 10.1186/s11671-016-1651-y

**Published:** 2016-10-01

**Authors:** Zhihua Li, Haixia Miao, Ying Fu, Yuxiang Liu, Ran Zhang, Bo Tang

**Affiliations:** Key Laboratory of Molecular and Nano Probes, College of Chemistry, Chemical Engineering and Materials Science, Collaborative Innovation Center of Functionalized Probe for Chemical Imaging in Universities of Shandong, Shandong Normal University, Jinan, 250014 China

**Keywords:** Hydrion rivalry aided by ultrasonic, NaYF_4_:Yb,Er, Bioimaging, Nanoprobe

## Abstract

A novel method of fabricating water-soluble bio-probes with ultra-small size such as NaYF_4_:Yb,Er (18 nm), NaGdF_4_:Yb,Er (8 nm), CaF_2_:Yb,Er (10 nm), PbS (7 nm), and ZnS (12 nm) has been developed to provide for the solubility switch of nanoparticles from oil-soluble to water-soluble in terms of hydrion rivalry aided by ultrasonic. Using NaYF_4_:Yb,Er (18 nm) as an example, we evaluate the properties of as-prepared water-soluble nanoparticles (NPs) by using thermogravimetric analyses (TGA), Fourier transform infrared spectroscopy (FTIR), zeta potential (*ζ*) testing, and 1H nuclear magnetic resonance (^1^HNMR). The measured *ζ* value shows that the newly prepared hydrophilic NaYF_4_:Yb,Er NPs are the positively charged particles. Acting as reactive electrophilic moiety, the freshly prepared hydrophilic NaYF_4_:Yb,Er NPs have carried out the coupling with amino acids and fluorescence labeling and imaging of HeLa cells directly. Experiments indicate that the method of hydrion rivalry aided by ultrasonic provides a simple and novel opportunity to transform hydrophobic NPs into hydrophilic NPs with good reactivity, which can be imaging some specific biological targets directly.

**Electronic supplementary material:**

The online version of this article (doi:10.1186/s11671-016-1651-y) contains supplementary material, which is available to authorized users.

## Background

In recent years, fluorescent label materials focus on the up-conversion (UC) phosphors represented by NaYF_4_:Yb,Er owing to their unique properties of emission light at shorter wavelengths (visible and near-infrared) after excitation in the near-infrared. Compared with conventional fluorescent materials, such as rhodamine, fluorescein, isothiocyanates, cyanine dyes [1, 2], and quantum dots [3–7], the luminous feature of up-conversion nanoparticles (UCNPs) dramatically reduces background autofluorescence since endogenous fluorophores are not excited by the longer excitation wavelengths, which results in high target-to-background ratios [8, 9], minimizing photodamage [10] and good tissue penetration [8, 9, 11–13]. UCNPs have become a kind of highly promising fluorescence labeling materials for biological applications.

Acting as bio-probes, the prepared UCNPs should meet some requirements of high fluorescent intensity [9, 14], small diameter [15], water-soluble [16, 17], low cytotoxicity [15, 18–21], etc. So, the control synthesis of UCNPs becomes to be crucial and attracts considerable interest in recent years [22–29]. To date, employing oleic acid (OA, C_17_H_33_COOH) to act as reaction media to obtain UCNPs with controlled size and morphology has been still a popular synthesis route [30–33]. Despite going well, the inherent shortcomings of OA layer covered on the as-prepared UCNP surface may prevent widespread use of UCNPs in practical biological settings. It is known that the OA layer covered on the surface of as-prepared UCNPs not only decreases the fluorescence intensity of UCNPs [16, 18] but also hinders their bio-application directly because of the water-soluble environment of biological fluids, namely, it is the necessary prerequisite to modify the surface of these NPs before applying into biomedical science. The advantages of surface functionalization of these NPs are not only to render them reasonably water-soluble and biocompatible but also to provide active sites for subsequent coupling with biological or chemical moieties [34–41]. So far, many attempts have been made to transform the oil-soluble OA-capped UCNPs into water-soluble, such as polymer capping [42, 43], surface silanization [44], OA-capped NaYF_4_:Yb,Er NP coating by hydrophilic group [45, 46], ligand exchange [47], ligand oxidation [48–50], hydrochloric acid [16], and layer-by-layer method [51]. Unfortunately, all of the post-treatments are time-consuming and may lead to aggregation; furthermore, the surface modified UCNPs always have bigger size than that of before.

Recently, we have studied the relationships between coating layer (OA) and NaYF_4_:Yb,Er in detail and come up with an easily operated physical method, ultrasonic separation, to remove the oleate ligand from the surface of OA-capped NaYF_4_:Yb,Er NPs [52]. Unfortunately, the ultrasonic separation method is hardly applied to the particles whose sizes are smaller than 20 nm, because a smaller NaYF_4_ particle will have a greater surface-to-volume ratio than the bigger one, and therefore displays a much greater surface free energy, which induces the growth and aggregation under the function of ultra-sound treatment. For application in vivo imaging, the size of UCNPs should be less than 10 nm [15]. In general, particles with dimension of 6 nm in diameter are the renal excretion limit size [53], and these ultra-small particles are excreted via the kidney and mostly excreted through the liver and into the bile without significant metabolism [54]. Even if UCNPs can be made especially small, these particles may become very bigger after water-soluble modification by currently common method, and the size of particles cannot meet the requirements for acting as nanobiotag to apply to the intracellular tracking of biomolecules. Recognizing the importance of water-soluble post-treatment of OA-capped UCNPs and how to develop a facial way to achieve for the solubility switch of NPs from oil-soluble to water-soluble meanwhile keeping the size and optical properties unchanged after treatment is challenging.

As known to all, the surface of NaYF_4_:Yb,Er NPs synthesized in OA reaction medium exists a fair amount of electron-poor metal atoms Y, which can coordinate with the carboxy groups of oleate anions. Considering OA is a weak acid, H^+^ will bond with oleate anions (C_17_H_33_COO^−^) easily if H^+^ is added into the above solution. Or rather, the positively charged NaYF_4_:Yb,Er NPs coming from electron-poor metal atoms Y and H^+^ have competitive reaction with C_17_H_33_COO^−^ in the solution. Considering “ultrasonic separation” also works well, it is expected that removing the oleate ligand from the surface of OA-capped NPs will be much easier by using of hydrion rivalry aided by ultrasonic.

Herein, we present a universal protocol, hydrion rivalry aided by ultrasonic, to remove the oleate ligand from the surface of OA-capped NPs. OA-capped ultra-small NPs, including NaYF_4_:Yb,Er (18 nm), NaGdF_4_:Yb,Er (8 nm), CaF_2_:Yb,Er (10 nm), PbS (7 nm), and ZnS (12 nm), act as examples to illustrate the transformation from oil-soluble NPs to water-soluble NPs. Considering after the removing of negatively charged oleate from the surface of NaYF_4_:Yb,Er, the newly prepared OA-free NaYF_4_:Yb,Er NPs may carry some positive charges, which will turn into reactive electrophilic moiety. We test the zeta potential of the freshly prepared water-soluble NaYF_4_:Yb,Er NPs and demonstrate the newly prepared OA-free NaYF_4_:Yb,Er NPs how to couple with amino acids and mark HeLa cells directly.

## Methods

### Materials

All of the commercially available reagents are purchased and unpurified. Rare-earth oxide (Y_2_O_3_, Yb_2_O_3_, Er_2_O_3_, Gd_2_O_3_, 99.9 %, Sigma), dichloride (CaCl_2_, PbCl_2_, ZnCl_2_, 99.9 %, Sigma), Na_2_S (99.9 %, Sigma), oleic acid (OA; 90 %, Sigma), 1-octadecene (ODE; >90 %, Sigma), absolute ethanol (>99.5 %, Sigma), cyclohexane (>90 %, Sigma), and rare-earth chlorides (LnCl_3_·6H_2_O, Ln = Y, Yb, Er, Y:Yb:Er = 80:18:2 in molar ratio) are prepared by ourselves.

### Synthesis of OA-Capped NaYF_4_:Yb,Er NPs

At first, the molar ratio of Er_2_O_3_, Yb_2_O_3_, and Y_2_O_3_ is 2 %:18 %:80 %, which are mixed and dissolved in hydrochloric acid solution and heated to form a clear solution. The above solution is evaporated and crystallized to obtain rare-earth chlorides (LnCl_3_). Second, 1 mmol dried LnCl_3_ with 6 ml of OA and 14 ml of ODE are added into a three-necked flask. The mixture is stirred and heated under vacuum to 150 °C to form a homogeneous solution A and then cooled down to room temperature. Then, another mixed transparent solution B (0.1 g NaOH, 0.148 g NH_4_F, and 10 ml of methanol) is added into solution A and then heated to 100 °C for 10 min. After methanol is evaporated thoroughly, the mixture is heated to 331 °C under nitrogen atmosphere for 60~90 min. Finally, the OA-capped NaYF_4_:Yb,Er NPs are obtained after the precipitate is centrifuged and washed with ethanol/cyclohexane (*v*/*v* = 1:1) for three times, which can be easily redispersed in various nonpolar organic solvents (e.g., hexane, cyclohexane, toluene).

### Synthesis of Oleate-Capped and OA-Free CaF_2_:Yb,Er, PbS, ZnS, and NaGdF_4_:Yb,Er Nanocrystals

The detailed experimental procedural is in the Additional file 1.

### Fabrication of Ligand-Free NPs

The as-obtained OA-capped NPs (0.5 g) is added into 10 ml ethanol, then adjusting the pH value of the solution to 4.5 by using diluted hydrochloric acid (0.1 mol/l). Simultaneously, the mixture is stirred vigorous and ultrasonic (power outlet 25–50 W) for 10~30 min, and then, the precipitate is separated by centrifugation. Finally, the hydrophilic NPs were washed with deionized water for two times and dried under vacuum at 60 °C.

### UCNPs Coupling with Amino Acid

Fifty-milligram amino acid is added into 10-ml water, and the mixture is stirred to form a homogeneous and transparent solution. Then, 20 mg of new-UCNPs (18 nm) is added into the above solution and vigorously stirred for overnight at 25 °C. The complex of amino acid UCNPs is obtained by centrifugation.

### Cell Culture, Fluorescent Labeling, and Imaging

The HeLa cells are cultured (at 37 °C, 5 % CO_2_) on glass chamber slides in RPMI 640 medium containing 10 % fetal bovine serum and 1 % penicillin/streptomycin overnight in a culture box (Heraeus BB16UV). The cells are gently washed three times with PBS and blocked in PBS containing 1 % bovine serum albumin (BSA) for 20 min at 4 °C. Then, HeLa cells are incubated with new-UCNPs (18 nm, 10 μg/ml) at 4 °C for 0.5–24 h. Prior to imaging, the live cells are washed thoroughly with PBS to remove any unbound reagents. Cell imaging is performed on a Leica DMIL inverted fluorescence microscope (with a ×40/0.5 objective) equipped with a 980-nm NIR laser and a Nikon digital camera.

### X-ray Diffraction

The samples are characterized by X-ray powder diffractometer (XRD) through using a Brucker D8-advance X-ray Diffractometer with Cu **K**α radiation (*λ* = 1.5418 Å); the operation voltage and current are, respectively, 40 kV and 40 mA. The 2*θ* range scan is swept from 10° to 70° in 0.02° steps with a count time of 0.2 s.

### TEM

Particle sizes and shapes are characterized by an H-7650 (HITACHI, Japan) low- to high-resolution transmission electron microscope (HRTEM) operated at 100 kV. Samples are prepared by drying a drop of nanocrystal dispersion in cyclohexane/toluene (1/1) or ethanol on amorphous carbon-coated copper grids.

### TGA

Thermogravimetric analyses are recorded on a TA Instrument SDT 2960 simultaneous DTA-thermogravimetric analyses (TGA) at a heating rate of 10 °C/min under N_2_.

### FTIR

The IR spectrum is obtained by using Brucker TENSOR Infrared Spectrometer.

### NMR

The NMR measurement is carried out on Bruker BioSpin GmbH Spectrometer.

### Zeta Potential

The zeta potential measurements are carried out on a Zeta PALS zeta potential analyzer (Brookhaven Instruments Corporation) at room temperature.

### Confocal Fluorescence Imaging of Incubated Living Cells

UCNP-labeled HeLa cells are characterized by a modified Olympus FV1000 laser scanning confocal microscope equipped a continuous-wave (CW) NIR laser operating at 980 nm (Connet Fiber Optics, China). A ×60 oil-immersion objective lens is used to carry out the FL imaging of UCNP-labeled HeLa cells. The emission of active UCNPs is collected at 540 ± 20 and 660 ± 20 nm under the 980-nm CW laser excitation.

## Results and Discussion

### Mechanism of Removing Oleate Ligand from the Surface of NaYF_4_:Yb,Er NPs

During the synthesis of NPs, OA acts as reaction media and takes part in the reaction. Before the reaction, OA reacts with Ln^3+^ ions to form Ln(OA)_3_, which renders LnCl_3_ disperses into the reaction system to ensure the homogeneous nucleation in the reaction. After the reaction, the carboxy group of oleate anions act as electron donors to coordinate with the rear earth ions possessing electron-poor and unsaturated bonding located at the surface of NaYF_4_:Yb,Er, which leads to a layer of OA covered on the surface of as-prepared NaYF_4_:Yb,Er. The bonding force between oleate and rear earth ions is weaker than that of normal chemical bond, which lies between physisorption and chemisorption, and can be separated by high-power ultrasonic wave [48]. However, the ultra-small particles, especially for the particles with their size less than 20 nm, have greater surface free energy, which always aggregate when using relatively high ultrasonic power to get rid of the OA layer from their surface. Therefore, how to eliminate the OA layer covered on the NaYF_4_:Yb,Er NPs under low ultrasonic power is an intractable problem.

**Fig. 1:**
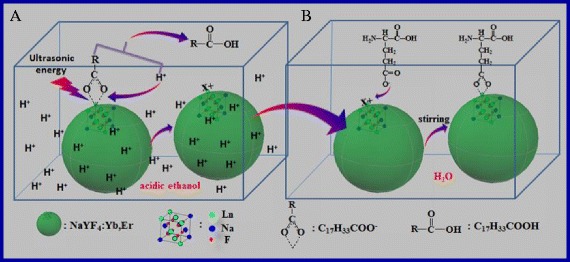
The schematic diagram of oleate ligand removed from the surface of NaYF_4_:Yb,Er NPs in the acidic ethanol (**a**) and the water-soluble new-UCNPs coupled with amino acid (**b**) in aqueous solution

As stated above, the surface of the as-synthesized NaYF_4_:Yb,Er NPs has a certain amount of positive charge, which can absorb the electronegative ions such as oleate to form OA-capped NaYF_4_:Yb,Er NPs. If H^+^ is introduced into the disperse system, the electrophilic substitution will occur that H^+^ replaces the positively charged NPs of NaYF_4_: Yb, Er to form a more stable weak acid, namely, oleic acid. Bogdan et al. carry out the transformation of oil-soluble to water-soluble NaYF_4_:Yb,Er NPs by using this principle [16]. Unfortunately, their experiments are a time-consuming process with very low yield. In our experiments, we adopt ultrasonic separation under weak acid to achieve the removing OA from OA-capped NaYF_4_:Yb,Er NPs. The mechanism of removing oleate ligand from the surface of NaYF_4_:Yb,Er NPs by using the way of hydrion rivalry aided by ultrasonic is showed in Fig. 1a. The H^+^ ion will attack the conjunction of the carboxyl and the rear earth atom of NaYF_4_:Yb,Er NPs, that is electrophilic substitution, namely, H^+^ ions and Ln^x+^ of NaYF_4_:Yb,Er NPs compete against each other to combine with carboxyl groups. What is more, H^+^ ions’ combination with oleate becomes much easy owing to the assistance of low-power ultrasonic; meanwhile, there is no agglomerative phenomenon for the ultrafine particles of NaYF_4_:Yb,Er (less than 20 nm). The formation of weak electrolyte, C_17_H_33_COOH, also promotes the reaction process.

### The Characterization of Samples

In our experiments, NaYF_4_:Yb,Er (18 nm), NaGdF_4_:Yb,Er (8 nm), CaF_2_:Yb,Er (10 nm), PbS (7 nm), and ZnS (12 nm) have been synthesized to testify the removing OA effect by using the method of hydrion rivalry aided by ultrasonic (Additional file 1). Herein, the as-prepared NaYF_4_:Yb,Er (18 nm) acts as a typical example to demonstrate our research.

**Fig. 2:**
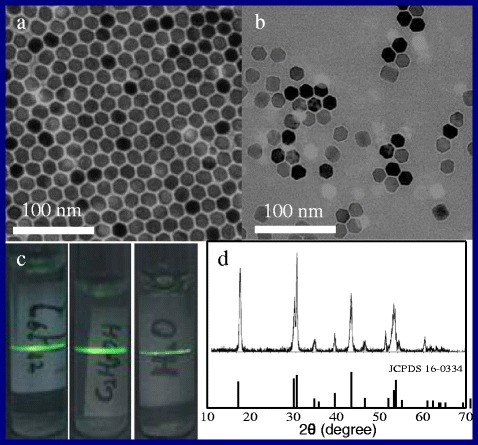
**a**–**d** The TEM images, the digital pictures, and XRD spectra of as-prepared OA-capped NaYF_4_:Yb,Er NPs and OA-free NaYF_4_:Yb,Er NPs, respectively

The transmission electron microscopy (TEM) images, the digital pictures, and XRD spectra of as-prepared NaYF_4_:Yb,Er NPs and OA-free NaYF_4_:Yb,Er NPs are shown in Fig. 2. As can be seen, the OA-capped NaYF_4_:Yb,Er NPs with the size of 18 nm are dispersed well in cyclohexane (1 mg/ml) (Fig. 2a). Figure 2b shows that the OA-capped NaYF_4_:Yb,Er treated by using ultrasonic power (40 W) under weak acid (pH ≈ 4) for 15 min can easily disperse in ethanol (1 mg/ml). The size of particles is approximately 18 nm with polyhedron morphology, and their size and shape of the samples have almost unchanged after post-treatment (Fig. 2b). Figure 2c shows the digital pictures of three clear solutions (OA-capped NaYF_4_:Yb,Er dispersed in cyclohexane, OA-free NaYF_4_:Yb,Er dispersed in ethanol and deionized water, respectively) radiated by 980 nm laser, all of the solution concentration here is 1 mg/ml. Figure 2d depicts the XRD curve of the as-prepared NaYF_4_:Yb,Er crystallizes with the β-phase of NaYF_4_, all of the diffraction peaks are lined with the data of JCPDS No.16-0334. The calculated size of the as-prepared particles is 18.2 nm according to the Debye-Scherrer equation, which agrees with the result from the TEM study.

**Fig. 3:**
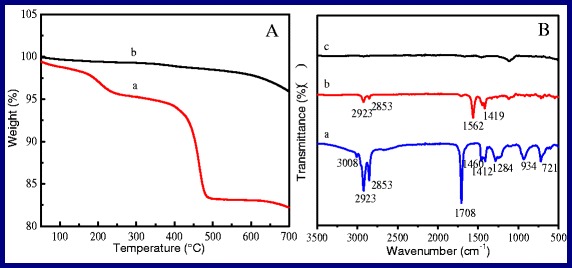
**a** The TGA curves of NaYF_4_:Yb,Er NPs. *a* OA-capped samples, *b* OA-free samples. **b** FTIR absorption spectra of as-prepared NaYF_4_:Yb, Er NPs before and after removing OA layer. *a* OA, *b* OA-capped samples, *c* OA-free samples

Figure 3a shows the TGA studies of the OA-capped NaYF_4_:Yb,Er NPs and OA-free NaYF_4_:Yb,Er NPs, respectively. Being synthesized in the assistance of OA surfactant, the surface of as-prepared NaYF_4_:Yb,Er has been covered a layer of oleate, namely, OA-capped. Therefore, the measured curve of OA-capped NaYF_4_:Yb,Er NPs shows two transformation points located at about 225 and 475 °C, respectively (Fig. 3a). The first weight loss stage is about 4.3 wt.% in the range of 30–250 °C which comes from the water loss. The second weight loss is 12.5 wt.% in the range of 250–600 °C, which is attributed to the combustion of the OA layer attached on the surface of sample. After samples are processed by using the method of hydrion rivalry aided by ultrasonic, the total weight loss is only about 2.2 wt.% and much lower than that of the untreated sample (4.3 + 12.5 %), which indicates that the weight loss of 2.2 wt.% is ascribed to the water loss, and the OA layer has been removed from the surface of as-prepared NaYF_4_:Yb,Er NPs.

The measured FTIR absorption spectra of as-prepared NaYF_4_:Yb,Er NPs before and after removing OA layer are showed in Fig. 3b. The FTIR spectrum of oleic acid is measured acting as the comparison experiment, which is depicted as curve a. For oleic acid, the specific absorbing peak of the alkene stretchings (C=C) and the asymmetric and symmetric stretching vibrations of the methylene (CH_2_) group are located at 3008 and 2923 and 2853 cm^−1^, respectively. The C=O stretching vibration mode and the O–H stretching vibration mode of the carboxyl group are located at 1708 and 934 cm^−1^, respectively. The curve b is the FTIR spectrum of OA-capped NaYF_4_:Yb,Er NPs, the specific absorbing peak of the alkene stretchings (C=C), and the asymmetric and symmetric stretching vibrations of the methylene (CH_2_) group located at 2923 and 2853 cm^−1^ separately with remarkable clarity in the spectrum. The absorption peaks of 1562 and 1419 cm^−1^ associate with the asymmetric and symmetric stretching vibrations of the carboxylic group (–COO^−^), respectively, instead of the peak at 1708 cm^−1^, which attributes to the electrostatic attraction and chemical adsorption between the Ln^3+^ ions of the surface of OA-capped NaYF_4_:Yb,Er NPs and –COO^−^ groups of the oleic acid. The results are lined with the previous reports [16, 55]. In Fig. 3c, the featured absorption peak of carboxyl at 1555 and 1459 cm^−1^ band cannot be found, which confirms that the sample c is OA-free NaYF_4_:Yb,Er NPs.

**Fig. 4:**
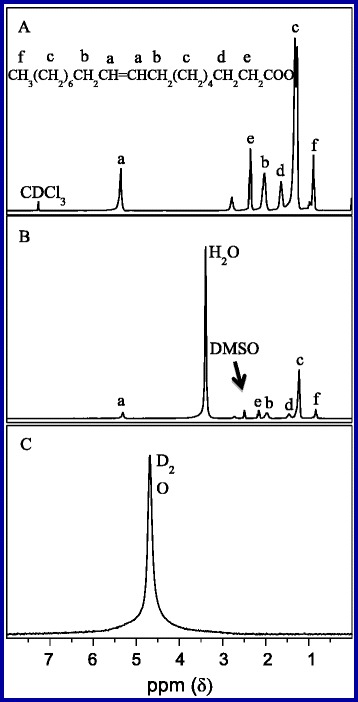
^1^HNMR spectra of three samples. **a** OA dispersed in CDCl3. **b** OA-capped samples dispersed in DMSO. **c** OA-free samples dispersed in D_2_O

Figure 4 shows the ^1^HNMR spectra of oil-soluble and water-soluble NaYF_4_:Yb,Er NPs, respectively. Figure 4a–c is the ^1^HNMR curves of OA dispersed in CDCl_3_, OA-capped NaYF_4_:Yb,Er NPs dispersed in DMSO, and OA-free NaYF_4_:Yb,Er NPs dispersed in D_2_O, respectively. As can be seen, all of the characteristic peaks of OA are one-to-one correspondence between sample A and sample B, which illustrates that the NaYF_4_:Yb,Er NPs are coated by OA. In Fig. 4c, the peak at 4.69 ppm which attributes to H_2_O from D_2_O ^1^HNMR spectra confirms that OA is removed from the surface of the NPs.

**Fig. 5:**
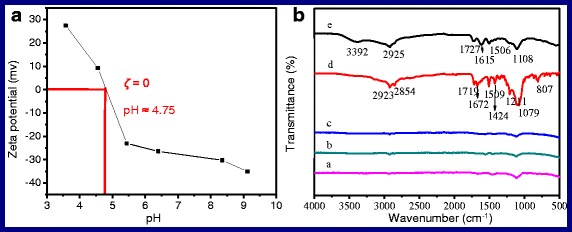
**a** The curve of the zeta potentials of OA-free NaYF4:Yb,Er NPs at different pH values. **b** The FTIR spectra of the complexes of amine acid coupling with NaYF4:Yb,Er NPs. *a* new-NaYF4:Yb,Er, *b–e* new-NaYF4:Yb,Er coupled with glycine, threonine, glutamic acid, and lysine, respectively

The value of zeta potential (*ζ*) is an important parameter, which indicates the stability condition of a colloidal system and how about the dispersed state of the NPs for testing in the solution. Lü et al. reports that the colloidal system with a zeta potential of ±25 mV will become stable where there is absence of coagulation [56]. Figure 5a shows the curve of the zeta potentials of OA-free NaYF_4_:Yb,Er NPs at different pH values. The solution of 0.1 mol/l PBS (pH = 7.4) acts as solvent to disperse the samples, which is prepared by using 0.1 mol/l Na_2_HPO_4_ and KH_2_PO_4_, HCl (0.1 mol/l) and NaOH (0.1 mol/l) which are used to adjust the pH value of the solution. As can be seen, the value of zeta potentials of the solution decreases gradually from +27.38 to −35.14 mV with the increase of pH value. At the point of zero charge, the pH value is 4.75, which corresponds to the PI isoelectric point. The value of zeta potentials of the OA-free NaYF_4_:Yb,Er NP solution is about −27 mV when pH equals to 7.4 (physiological solution). As a result, the testing OA-free NaYF_4_:Yb,Er NPs can disperse into aqueous solution steadily. In other words, we carry out the transformation from oil-soluble NPs to water-soluble NPs successfully.

As analysis in the mechanism section, the surface of the freshly synthesized NaYF_4_:Yb,Er NPs exists unsaturated bonding of rear earth ions, which coordinates with the carboxy group of oleate anions to form OA-capped NaYF_4_:Yb,Er NPs. So, the freshly prepared OA-free NaYF_4_:Yb,Er NPs turn into positively charged particles after removing the covered layer, which can be predicted that the high activity dangling bond existed on the surface of particles will be neutralized by their storage environment if they are placed in the air for a long time and finally result in the loss of positive charge from the surface of the particles. As known, positive or negative of zeta potential represents the nanoparticles with what kind of charge according to the definition of zeta potential, normally, *ζ* > 0 means the positively charged particles, whereas the particles carry a negative charge. Herein, we prepare two samples, one is the freshly prepared OA-free NaYF_4_:Yb,Er NPs (new-UCNPs) and the other is OA-free NaYF_4_:Yb,Er NPs which are placed in the air for 2 months (aged-UCNPs). The measured zeta potential of new-UCNPs is +32.96 mV under aqueous solution of pH = 6, which indicates that the new-UCNPs are positively charged particles. Though the aged-UCNPs also have excellent water-solubility, they are negatively charged particles because its measured zeta potential is −29.50 mV. The results are lined with our prediction. In consideration of the positively charged new-UCNPs possess electrophilicity, it is possible that the freshly prepared OA-free NaYF_4_:Yb,Er NPs can react directly with negatively charged centers on biomolecules and form UCNP-biomolecule adducts.

### UCNPs Couple with Amino Acid

**Fig. 6:**
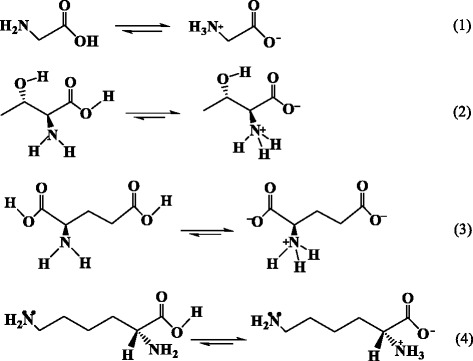
The solubility equilibrium equations of alpha-amino-beta-hydroxybutyric acid, glycine, glutamic acid, and l-lysine in the aqueous solution, respectively

Amino and carboxy are electron-donating group, which can attract with positively charged ions, such as the unsaturated bonding of rear earth ions exist on the surface of the new-UCNPs to form adducts. Herein, alpha-amino-beta-hydroxybutyric acid [CH_3_CH(OH)CH(NH_2_)COOH], glycine [NH_2_CH_2_COOH], glutamic acid [HOOCCHNH_2_(CH_2_)_2_COOH], and l-lysine [H_2_NCH_2_CH_2_CH_2_CH_2_CH(NH_2_)COOH] are employed to couple with the aging-UCNPs (18 nm) and new-UCNPs (18 nm), respectively. The FTIR spectra of the complexes of amine acid coupling with NaYF_4_:Yb,Er NPs are shown in Fig. 5b, respectively. As can be seen, curve a is the FTIR spectrum of new-UCNPs. Curves b and c (corresponding to the products of new-UCNPs react with glycine and threonine, respectively) have the same profile with curve a and absence all of the characteristic peaks of amino acid, which indicates that new-UCNPs cannot couple with glycine and threonine. For the curves d and e, the typical absorption peaks located at 2923, 2854, and 2925 cm^−1^ come from the asymmetric and symmetric stretching vibrations of the C−H bond (–CH_2_), respectively [57]. The peaks of 1719 and 1727 cm^−1^ correspond to the typical amide carbonyl absorption band. The absorption bands located at 1615 and 1672 cm^−1^ are attributed to the N−H bending mode of amino group (−NH_2_) [58]. The peak at 3392 cm^−1^ is attributed to the stretching vibration of amine groups. The peaks of 1424, 1506, and 1509 cm^−1^ belong to the stretching vibrations of the C−N bond. The absorption bands of 1211 cm^−1^ and (1079, 1108 cm^−1^) belong to the C–N and C–O bond, respectively. The peaks at 807 cm^−1^ are attributed to the C–O–Y vibration [56]. All of the signals illustrate that the freshly prepared water-soluble NaYF_4_:Yb,Er NPs have coupled with glutamic acid and lysine successfully. Why glutamic acid and lysine? Figure 6 shows the solubility equilibrium equations of the above amino acids in the aqueous solution.

As known, adjacent amino and carboxy of amino acid can form into inner-salt in the aqueous solution. So, the group of −NH_3_^+^ will act as steric hindrance to hinder the electron-donating group, carboxylate ion, to couple with positively charged ions, and leads to CH_3_CH(OH)CH(NH_2_)COOH and NH_2_CH_2_COOH seldom react with new-UCNPs finally.

For HOOCCHNH_2_(CH_2_)_2_COOH and H_2_NCH_2_CH_2_CH_2_CH_2_CH(NH_2_)COOH, such as in Fig. 6 Eq. (3) and (4), one end of the molecules forms inner-salt, the existence of −NH_3_^+^ acting as steric hindrance will hinder the coupling between carboxyl and UCNPs; the other end of the molecules keeps the strong nucleophilicity group, carboxyl (Fig. 6 formula 3) and amino (Fig. 6 formula 4). Therefore, HOOCCHNH_2_(CH_2_)_2_COOH and H_2_NCH_2_CH_2_CH_2_CH_2_CH(NH_2_)COOH can couple with newly UCNPs directly, as shown in Fig. 1b. In Fig. 5, we also notice that curve d has a higher signal of amino acid than that of curve e, which indicates that the effect of HOOCCHNH_2_(CH_2_)_2_COOH coupling with UCNPs is better than that of H_2_NCH_2_CH_2_CH_2_CH_2_CH(NH_2_)COOH. The reason may be that carboxyl nucleophilic performance is better than that of amino.

### Fluorescence Labeling and Imaging of HeLa Cells

**Fig. 7:**
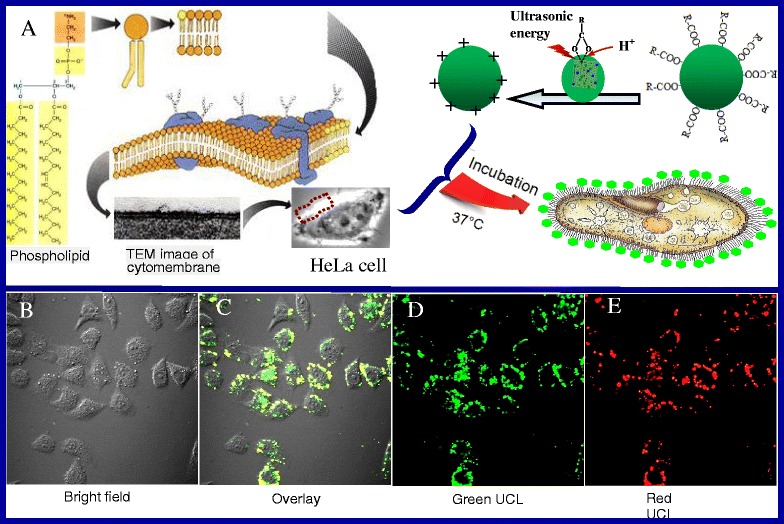
**a** The sketch map of bio-labeling procedural of HeLa cells. **b**–**e** The confocal luminescence images of HeLa cells incubated with UCNPs at 37 °C for 16 h, respectively

It is common sense that the cell membrane is always negatively charged, so the cationic particles tend to binding negatively charged groups on the cell surface (e.g., sialic acid, phosphate group) [59]. As positively charged particles have the greatest efficiency in cell-membrane penetration and cellular internalization, they form the primary platform as synthetic carriers for drug and gene delivery [60–62]. As mentioned, the new-UCNPs are active particles with positive charges owing to the dangling bond of Ln^3+^ (Ln = Y, Yb, Er) on the surface of particles, which should be able to bind with the negatively charged groups on the surface membranes of HeLa cells. Figure 7a shows the structure schematic diagram of HeLa cells and its bio-labeling procedure. The cytomembrane is comprised of phospholipid bilayers, as shown in Fig. 7, which have numerous negatively charged phosphate groups on the surface of phospholipid. If the new-UCNPs are incubated with HeLa live cells in physiological conditions, the coupled reaction will occur, and the new-UCNPs are thus linked to the surface of the cells. As a result, HeLa cells are labeled by NaYF_4_:Yb,Er UCNPs.

To ascertain whether the new-UCNPs (18 nm) have been conjugated HeLa cells after incubation (C_UCNPs_ = 10 mg/ml) for 16 h at 37 °C, the cells are washed five times using PBS to get rid of the residual UCNPs thoroughly and then imaged using a confocal microscope equipped with a 980-nm NIR laser. Figure 7b–e shows the confocal luminescence images of new-UCNP-labeled HeLa cells. As is shown, the surrounding cells exhibit bright green and red UC fluorescence, confirming the attachment of the new-UCNPs conjugates on the surface of cells. The morphology and position of the cells in bright field and dark field overlap very well, showing stronger interactions between the positively charged UCNPs and the negative charged phosphate groups on the surface of phospholipid of cells. In addition, considering a long incubation time of HeLa cells with the high concentration of new-UCNPs, the labeled cells also keep considerable viability, which indicates that the new-UCNPs have good biocompatibility and low cytotoxicity. However, the biological environment is more complex, has different pH, and has the presence of proteins as well as other macrophages and nonspecific binding molecules that are everywhere in vivo model. For the consideration of our experiments just to have a specific target, the further applications in the biomedical field after addressing specificity and selectivity to target an analyte or a cell of interest should be done in the future.

## Conclusions

We present an effective method to carrying out the solubility switch of nanoparticles from oil-soluble to water-soluble in terms of hydrion rivalry aided by ultrasonic; furthermore, the method is suitable for the ultra-small particles. The measured zeta potential indicates that the newly prepared OA-free NaYF_4_:Yb,Er NPs have some positive charges, which is demonstrated to couple with amino acid and label and image the HeLa cells further. The biology experiment illustrates that the freshly prepared OA-free UCNPs can act as bio-probe for some specific target applications directly without any surface functional treatment.

## Additional file

Additional file 1: Figure S1. TEM images and XRD patterns of CaF_2_:Yb,Er, PbS, ZnS, and NaGdF_4_:Yb,Er NPs of removing OA layer before and after, respectively. (DOC 1386 kb)
