# Supplementary material for: Fabrication of NaYF4:Yb,Er Nanoprobes for Cell Imaging Directly by Using the Method of Hydrion Rivalry Aided by Ultrasonic
Source: Nanoscale Res Lett. 2016 Oct 1;11:441. doi: 10.1186/s11671-016-1651-y (PMC5045454; doi:10.1186/s11671-016-1651-y)
Supplement: Additional file 1: Figure S1. — TEM images and XRD patterns of CaF2:Yb,Er, PbS, ZnS, and NaGdF4:Yb,Er NPs of removing OA layer before and after, respectively. (DOC 1386 kb) [file 11671_2016_1651_MOESM1_ESM.doc]

**Additional file 1**

Zhihua Li*, Haixia Miao, Ying Fu, Yuxiang Liu, Ran Zhang, Bo Tang*

College of Chemistry, Chemical Engineering and Materials Science, Key Laboratory of Molecular and Nano Probes, Collaborative Innovation Center of Functionalized Probe for Chemical Imaging in Universities of Shandong, Shandong Normal University, Jinan, 250014, China

Corresponding Authors: [lizhihua2006@126.com,](mailto:lizhihua2006@126.com,) [tangbo@sdnu.edu.cn](mailto:tangbo@sdnu.edu.cn)

**Synthesis of Oleate-Capped** **CaF_2_:Yb,Er nanocrystals**

Mixed rare earth chlorides (2.5 mmol, CaCl_2_ + YbCl_3_ + ErCl_3_, the molar ratio of Ca:Yb:Er is 78：20：2 ) and NaOH (15 mmol) were added into the mixture of deionized water (4 ml), ethanol (4 ml) and oleic acid (10 ml). Then the mixture was stirred vigorously for 30 min, and then NaF (1 mmol) was added into the mixture. After stirring for another 10 min, the turbid liquid was transformed into autoclave (30 ml) and heated to 180 °C for 36 h. The final products were collected by means of centrifugation, and washed with ethanol for three times, which can disperse in cyclohexane easily (J. Am. Chem. Soc. 2009, 131, 14200–14201). The TEM image of as-prepared sample was showed in Figure SA_1_, the average size of CaF_2_:Yb,Er NPs was 10 nm.

**Synthesis of Oleate-Capped PbS and ZnS nanocrystals**

The synthesis procedural is as the same as that of the synthesis of CaF_2_:Yb,Er. The only difference is that the ions of Pb^2+^, Zn^2+^ and S^2-^ were instead of above mixed rare earth chlorides and NaF. The TEM images of as-prepared samples were showed in Figure SB_1_ and in Figure SC_1_, the average sizes of PbS and ZnS NPs were 7 nm and 12 nm, respectively.

**Synthesis of Oleate-Capped NaGdF_4_:Yb,Er** **naoparticles**

The synthesis procedural is as the same as that of the synthesis of NaYF_4_:Yb,Er (Please the manuscript, page 4: Synthesis of OA-capped NaYF_4_:Yb,Er NPs). The difference is the initial reactants, which is to substitute Y_2_O_3_ for Gd_2_O_3_. The TEM image of as-prepared sample was showed in Figure SD_1_, the average size of NaGdF_4_:Yb,Er NPs was 8 nm.

**Preparation of hydrophilic nanocrystals**

The as-obtained OA-capped NPs (0.1 g) is added into 2 mL ethanol, then adjusting the pH value of the solution to 4.5 by using diluted hydrochloric acid (0.1 mol/L). Simultaneously, the mixture is stirred vigorous and ultrasonic (power outlet: 25-50 W) for 10~30 min, and then the precipitate is separated by centrifugation. Finally, the hydrophilic NPs were washed with deionized water for two times and dried under vacuum at 60 °C.

The TEM images and XRD patterns of CaF_2_:Yb,Er, PbS, ZnS and NaGdF_4_:Yb,Er NPs of removing OA-layer before and after, respectively.


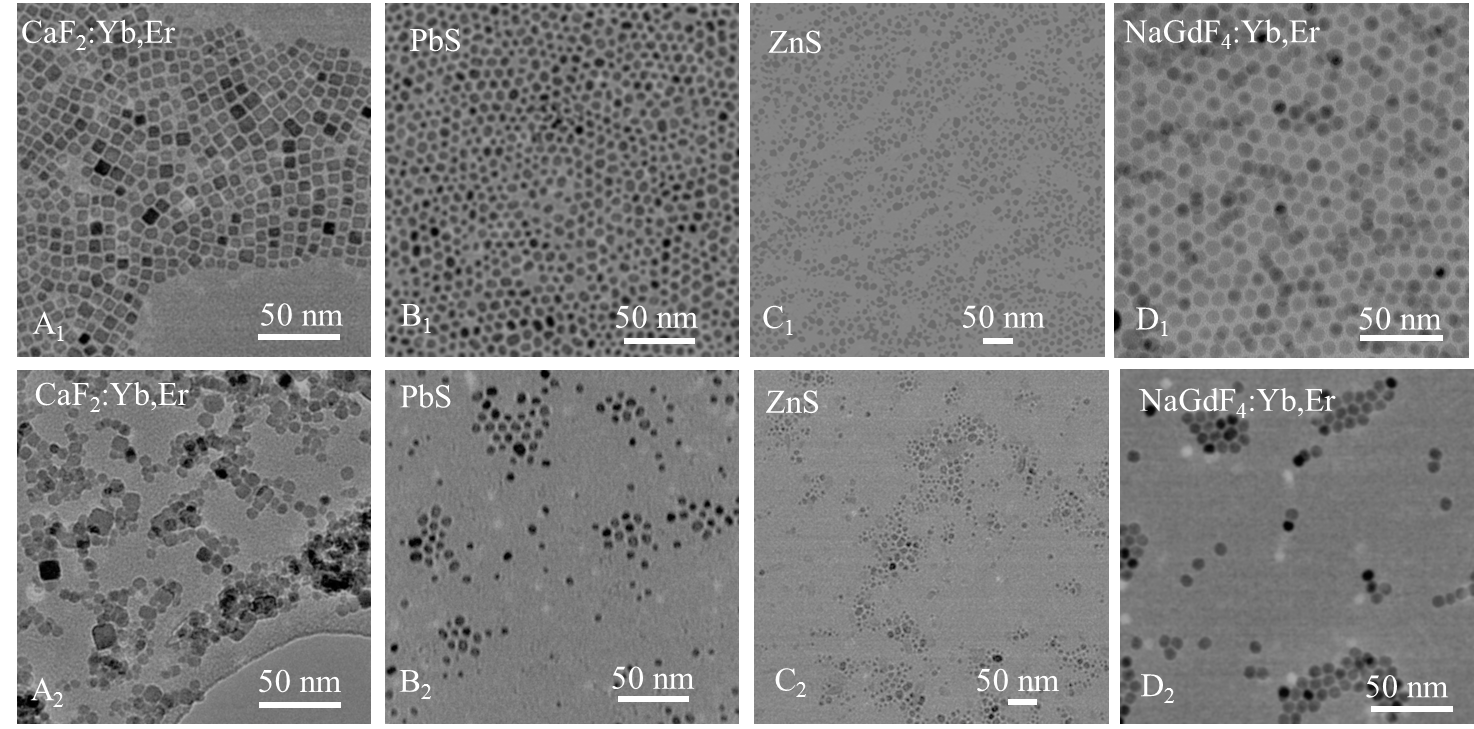


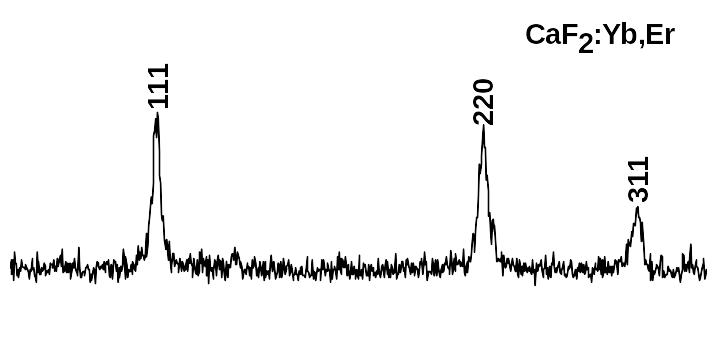

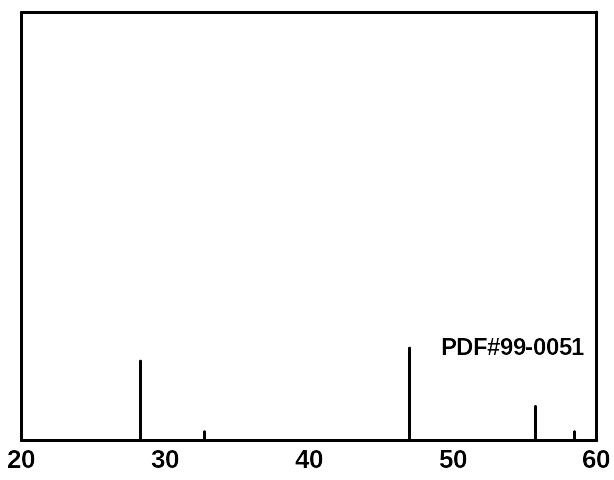


2θ (degree)


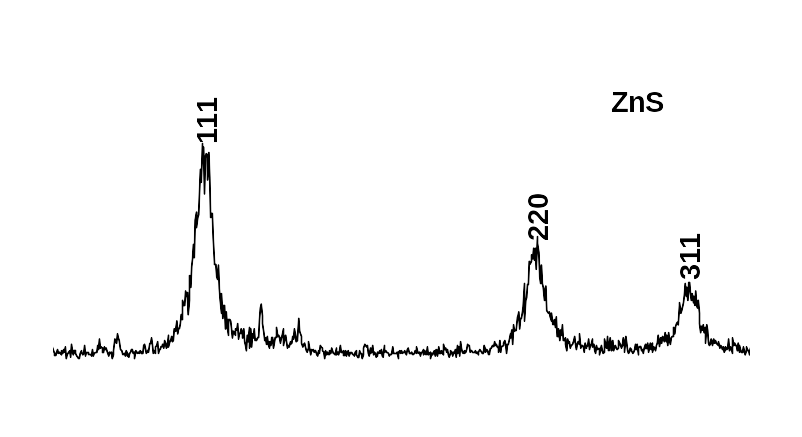

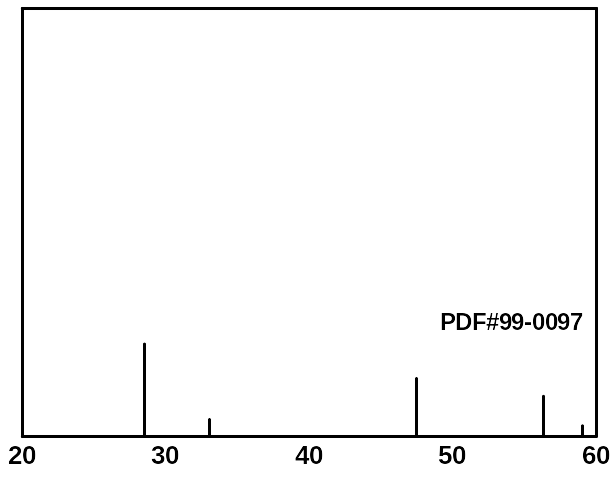


2θ (degree)


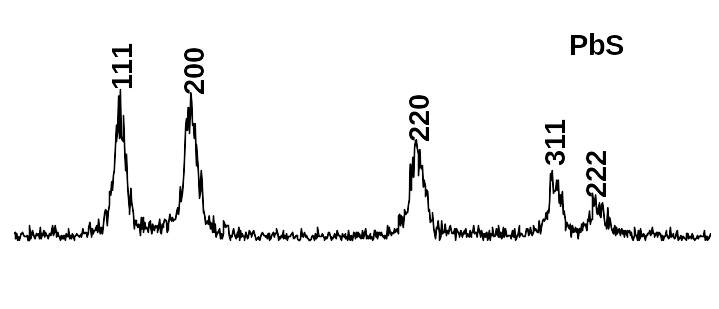

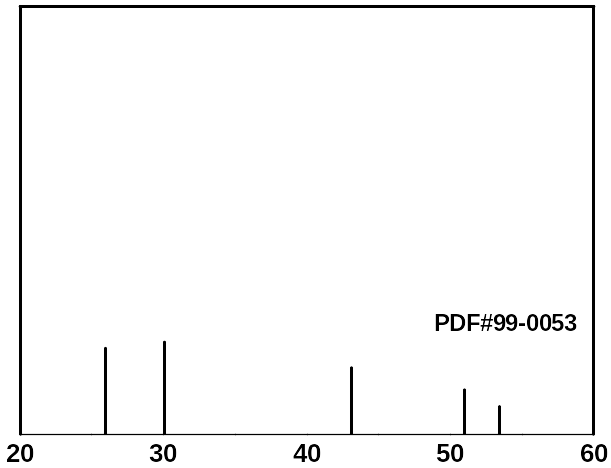


2θ (degree)

2θ (degree)

NaGdF_4_:Yb,Er

100

101

110

100

111

201

102

211

210

112

PbS

**Figure S1.** The TEM images and XRD patterns of CaF_2_:Yb,Er, PbS, ZnS and NaGdF_4_:Yb,Er NPs of removing OA-layer before and after, respectively. OA-capped NPs disperse into cyclohexane were showed in A_1_-D_1_, OA-free NPs disperse into ethanol were showed in A_2_-D_2_.
